# Consistency in boldness, activity and exploration at different stages of life

**DOI:** 10.1186/1472-6785-13-49

**Published:** 2013-12-07

**Authors:** Antje Herde, Jana A Eccard

**Affiliations:** 1 Department of Animal Ecology, University of Potsdam, Maulbeerallee 1, 14469 Potsdam, Germany; 2 Department of Animal Behaviour, University of Bielefeld, Morgenbreede 45, 33615 Bielefeld, Germany

**Keywords:** Animal personality, Behavioural type, *Microtus arvalis*, Common vole, Plasticity, Consistency, Repeatability

## Abstract

**Background:**

Animals show consistent individual behavioural patterns over time and over situations. This phenomenon has been referred to as animal personality or behavioural syndromes. Little is known about consistency of animal personalities over entire life times. We investigated the repeatability of behaviour in common voles (*Microtus arvalis*) at different life stages, with different time intervals, and in different situations. Animals were tested using four behavioural tests in three experimental groups: 1. before and after maturation over three months, 2. twice as adults during one week, and 3. twice as adult animals over three months, which resembles a substantial part of their entire adult life span of several months.

**Results:**

Different behaviours were correlated within and between tests and a cluster analysis showed three possible behavioural syndrome-axes, which we name boldness, exploration and activity. Activity and exploration behaviour in all tests was highly repeatable in adult animals tested over one week. In animals tested over maturation, exploration behaviour was consistent whereas activity was not. Voles that were tested as adults with a three-month interval showed the opposite pattern with stable activity but unstable exploration behaviour.

**Conclusions:**

The consistency in behaviour over time suggests that common voles do express stable personality over short time. Over longer periods however, behaviour is more flexible and depending on life stage (i.e. tested before/after maturation or as adults) of the tested individual. Level of boldness or activity does not differ between tested groups and maintenance of variation in behavioural traits can therefore not be explained by expected future assets as reported in other studies.

## Background

There has been increasing interest in consistent differences in individual behaviour across time and/or contexts. For example, animals of the same sex, weight, and population often differ consistently in their aggressiveness in different situations. This phenomenon has been referred to as behavioural syndromes [1-3] or animal personalities [4,5]. Behavioural syndromes are an attribute of populations and cover rank-order differences between individuals. A behavioural type, in contrast, describes the attributes of an individual and covers particular configurations of behaviours that one individual expresses [1]. The presence of behavioural syndromes or types and the connection between different behaviours in a variety of contexts mean that an individual’s behaviour is not infinitely flexible [6]. From an adaptive perspective, limited plasticity is unexpected because heterogeneous environments should favour the evolution of behavioural plasticity rather than behavioural consistency [7,8]. Meanwhile, personality and individual plasticity might also be linked [9]. For example, studies on laboratory mice and rats show that aggressive behaviour is related to the way animals cope with different situations. Non-aggressive males seem to be more flexible in their behaviour during environmental challenges compared to more aggressive males [10].

Although consistent differences in behaviour among individuals can be found in a wide range of species, there is still not much knowledge about the origin and the impact of personality or behavioural types. Some studies indicate strong genetic bases for behavioural syndromes [11,12], others suggest that maternal effects [13] might play a role, as well as life-history stage ad experiences of the individual [3,5,7]. In particular, prior experience can influence the behaviour of an individual immediately and also later in life. For example, threespined stickelbacks (*Gasterosteus aculeatus)* showed a stronger behavioural correlation between aggressiveness and boldness after they had been exposed to a predator [14]. Behavioural types affect the life-time reproductive success of an individual and can be understood as a component of its life history [6,15-18]. Therefore it is crucial to understand ontogenetic development of animal personality, whether and when during lifetime it develops, it becomes fixated, and how stable it is over an individual’s life time. Studies on consistency over time in birds [19,20] and insects [21-23] showed that stability and repeatability is variable for different behaviours and also for the species under investigation. Hence, it cannot generally be assumed that consistent individual differences in behaviour are stable throughout life and a validation for different behaviours, life stages and species is necessary.

While consistency is the fundamental part of the definition of behavioural syndromes, many terms were used for different types of consistency over time and over situations [24]. In this study we focussed on temporal consistency, especially differential consistency, and contextual generality. Differential consistency refers to ‘the extent to which scores for behaviour in a given context at a given time are correlated across individuals with scores for the same behaviour in the same context at a later time’ [13,24] and can also be called repeatability. We investigated the consistency over time in four behavioural tests (barrier-test, open-field, dark–light and nose-in-hole-test), and used common voles (*Microtus arvalis*) in different life stages as a model organism. We expect that the repeatability in behaviour of common voles is higher when interobservation interval is short, as it was shown for humans [25] and great tits (*Parus major*) [26,27]. It is more likely to test an animal in the same (e.g. reproductive) state within a short interobservation interval and the opportunity for developmental change is high when the time between two tests is long [28]. Here, we compared wild captured adult animals that were either tested twice within one week (‘adult short term’) or a second time after a period of three month (‘adult long term’). The maximum life span of voles (genera *Microtus)* is around 17 month [29] but due to massive predation all over the year and in every developmental state, individuals often die much earlier. Therefore, an interobservation interval of three month covers nearly a whole life span of a common vole.

Since maturation is a sensitive phase during individual development in many species, we further expected, that the behaviour of individuals tested before and after maturation would be less consistent compared to animals tested in the same time interval as fully developed adults. Changes in endocrinological and neuronal systems as well as new challenging environmental factors like novel habitats and unfamiliar conspecifics can affect this variation in behaviour [30,31].

Contextual generality refers to ‘the extent to which scores for behaviour expressed in one context are correlated across individuals with scores for behaviour expressed in one or more other contexts, when behaviour in all of the contexts is measured at the same age and time’ [13,24]. We compared the different latencies and activities measured in the four behavioural tests during one week in adult animals (short term adult), to get an impression of a possible relationships between those behaviours in common voles. We expect that measured latencies and activities were linked to each other as it has been found in other species (e.g. [10,32-35]). Possible behavioural syndrome axis in behaviour will be shown in a dendrogram as the output of a cluster analyses.

## Methods

### Study system

The common vole (*Microtus arvalis*) is a widespread fossorial rodent in Europe with a polygynandrous mating system. Females can share nests and form colonies with sisters and/or daughters during lactation [36,37]. Females can give birth to several litters with 2–8 pups per litter (mean 5.2) during one reproductive season [29]. During winter, male antagonism decreases and animals overwinter in mixed groups [38]. Weight of adult common voles can vary between 18 to 40 g [29]. Active phases are distributed evenly over day and night in a 2–3 hour circle with peaks in activity during twilight [39].

### Behavioural consistency over maturation

The experiments of the ‘over maturation’-part of this study were conducted between January and July 2008 in the facilities of the Department of Animal Behaviour of the University of Bielefeld, Germany (52°02’10.72”N, 8°29’23.12”O). We used 17 laboratory-born common voles (9 males, 8 females; Table 1), bred from originally wild voles that were trapped in 2007 in Bielefeld. Animals were kept in breeding rooms with light adjusted to seasonal day length Rooms were not heated except for frost periods to prevent freezing of water bottles. Animals were kept singly after being weaned from their mothers in standard makrolon cages (Ehret GmbH Germany, Typ III: 42 cm × 27 cm × 16 cm), containing wood shavings, hay and paper rolls for shelter. Water and food pellets (Altromin international, Germany; standard laboratory mice food) were available *ad libitum*. The first testing period started when animals were 62 ± 20 days old and immature (visual inspection for closed vagina or abdominal testes). Animals were habituated to the experimental room (20-23°C and artificial lighting) two hours before testing, and the barrier-test (description below) was conducted under direct observation. This procedure was repeated in a second testing period 90 days later when all animals had matured (open vagina or scrotal testes).

**Table 1:** Consistency over time in behaviour of common voles in four tests in three experimental groups

**Test**	**Variable**	**Definition**	**Mean ± SD**	**over maturation**			**adult - short term**			**adult - long term**		
				**N**	**r**_**s**_	**p**	**N**	**r**_**s**_	**p**	**N**	**r**_**s**_	**p**
Barrier	Latency	Latency to jump over barrier	44.51 ± 68.8	**17**	**0.699**	**0.01**	**168**	**0.409**	**<0.01**	48	0.122	1.00
			(466)									
	Activity	1-0 sampling every 10 sec.	19.43 ± 8.09	17	0.205	0.43	**151**	**0.441**	**<0.01**	**41**	**0.561**	**<0.01**
			(418)									
	Crossing frequency	No. of crossing barrier per minute	2.64 ± 2.94				**168**	**0.581**	**<0.01**	**48**	**0.636**	**0.03**
			(432)									
Open field	Latency unsafe zone	Latency to go in middle zone	132.19 ± 80.49				**157**	**0.388**	**<0.01**	47	0.229	1.00
			(408)									
	Activity	1-0 sampling every 10 sec.	19.96 ± 7.57				**164**	**0.543**	**<0.01**	**47**	**0.515**	**0.05**
			(422)									
	Time safe zone	1-0 sampling every 10 sec.	24.93 ± 5.51				**164**	**0.354**	**<0.01**	47	0.179	1.00
			(422)									
Dark–light	Latency into light	Latency to go in light compartment	90.22 ± 169.54				**164**	**0.572**	**<0.01**	25	0.28	1.00
			(378)									
	Time in light	Time spend in light zone [sec]	104.22 ± 161.9				**164**	**0.388**	**<0.01**	**25**	**0.737**	**0.01**
			(378)									
Hole	Latency one hole	Latency to find one hole	86.66 ± 65.36				**132**	**0.34**	**0.01**	10	0.091	1.00
			(284)									
	Latency four holes	Latency to find all four holes	237.32 ± 71.89				**132**	**0.372**	**<0.01**	10	0.119	1.00
			(284)									
	Number nose	No. of nose-in-hole events	10.63 ± 6.89				**132**	**0.558**	**<0.01**	10	-0.031	1.00
			(284)									

Mean ± standard deviation (SD) was calculated from all conducted tests. Number of conducted tests is given in parentheses. Spearman rank correlations coefficient (r_s_) was calculated between first and second testing. P-values were adjusted for multiple testing with Holm correction. Significant correlations are in bold (p < 0.05).

### Behavioural consistency in adulthood

The experiments on adult voles were conducted between April and November 2010 and February and September 2011 in the field station of the Department of Animal Ecology of the University of Potsdam, Germany (52°26’21.83”N, 13°00’44.14”O).

We captured 248 common voles with live traps (Ugglan special No2, Grahnab, Sweden) from meadows around Potsdam. Traps were always baited with rolled oats (as food) and apple (as water reserve) and were checked every 12 hours during trapping periods. Captured, adult voles were brought to the laboratory. Lactating females and juveniles were immediately released at the trapping side. Animals were housed singly at room temperature (15-25°C, changing with season) and natural seasonal photoperiod in same cage-conditions as described above immediately after trapping. Water and food pellets (ssniff V1594 R/M-H Ered II) were available *ad libitum* and the diet was enriched with carrots, potatoes and fresh grass. Testing phases started 3–6 weeks after the animals were captured and all pregnant-captured females had given birth and had weaned their young. Weanlings were released at the original trapping side of the mother.

In 2010, 168 adult common voles (88 males, 80 females; Table 1) were tested two times in four behavioural tests (description below) within one week to test the consistency of vole behaviour during a short period (group ‘short term adult’).

Forty-eight adult common voles captured in 2010 (8 males, 21 females) and 2011 (11 males, 8 females; Table 1) were used to test the consistency of the behaviour over 2–3 months (group ‘long term adult’). All animals were tested once in the barrier-test and the open-field (description below) 3–6 weeks after trapping. Afterwards, the animals were marked individually with a unique passive integrated transponder (‘PIT’; Trovan ID-100; 2.12 mm × 11.5 mm, 0.1 g) implanted at the neck. We found no evidence for negative effects of the implantation on the animals. Marked voles were transferred to 0.25 ha outdoor enclosures with natural vegetation and natural avian predation (enclosures were not netted) in groups of 8 animals (4 males, 4 females) per enclosure. After 5–6 weeks, we trapped the voles back from the enclosures. Voles were transferred to the laboratory again and were tested a second round in barrier-test and open-field 3–6 weeks later.

### Behavioural tests

For the behavioural testing, we modified standard laboratory tests that were originally used to test emotionality or fearfulness in mice and rats, which are now commonly used in studies on behavioural syndromes in other species [4]. We adjusted the set-ups of the barrier-test [40,41], open-field test [42] and dark–light-test [43] for the needs and skills of non-climbing, subterranean, wild-captured voles. In addition, we invented the nose-in-hole-test (similar to hole-board test in [33]; thereafter called hole-test) to investigate exploration behaviour. Variables that were tested reflect mainly boldness, exploration and activity of the tested animals. Tests were directly observed between 0800 and 1800 hours with a minimum of two hours rest for the animals between tests.

#### Barrier-test

A semi-transparent plastic box (45 cm × 22 cm × 25 cm) was divided into two equal compartments by a 4.5 cm high barrier (grey PVC). According to a pseudo-random schedule, the animal was placed in one of the compartments and the latency was measured until the animal crossed the barrier from one compartment to the other. If animals did not jump over the barrier within 5 minutes,% latency was set to the maximum of 300 seconds (2.58% of all performed tests). The activity of the animal was recorded every 10 seconds with instantaneous 1-0-sampling (max. 30 active samples). Additionally, the number of crossings was counted for all adult individuals (in 2010 and 2011). The variable ‘crossing frequency’ (crossings per minute during time interval left after substraction of latency) was calculated for the analyses.

#### Open-field

We used a round metal arena (1 m diameter, wall 35 cm high) as an open-field with a safe wall zone (20 cm wide) and an middle zone, that is known to be perceived as “unsafe” for small mammals [42]. The animal was placed in the middle of the arena in a tube. The tube was lifted and the test duration of 5 minutes started at the moment the vole reached the wall of the arena the first time. Latency to re-enter the “unsafe” middle of the arena was measured. If animals did not move to the unsafe zone within 5 minutes, latency was set to the maximum of 300 seconds (15.2% of all performed test). In addition, activity (max. 30 active samples) and time in the safe wall zone of the arena (max. 30 samples in the safe area) were recorded with instantaneous 1-0-sampling every 10 seconds.

#### Dark–light-test

A black plastic box (30 cm × 30 cm × 15 cm) with an entrance hole (4 cm × 5 cm) was placed upside down into a larger white plastic box (65 cm × 50 cm × 30 cm). The animal was placed in the black box and the latency to come out of the dark (‘latency into light’) and the time to go back (‘time in light’) in a maximum time of 10 minutes were measured. If animals did not leave the dark box within 10 minutes, latency was set to the maximum of 600 seconds (8.99% of all performed tests).

#### Hole-test

A vole was placed in the middle of a standard makrolon cage (like housing cage, without bedding) with 4 holes (1 cm diameter in 1 cm height), one in each corner. Over 5 minutes, the latency to first ‘nose-in-hole’-event, the latency to explore all 4 holes, and the total number of nose-in-hole-events (thereafter called ‘number holes’) were measured. If animals did not stick its nose in a hole within 5 minutes, latency was set to the maximum of 300 seconds (4.55% of all performed tests). The same was true if they did not discover all 4 holes within the test period (44.72% of all performed tests).

### Statistical methods

Many measured variables were distributed in a skewed manner (a few similar to bimodal distributions) rather than a normal one (Kolmogorow-Smirnow-test). Therefore, we mainly used non-parametric statistics. Comparisons between experimental groups were calculated with Kruskal-Wallis-test and accordingly between testing rounds with Wilcoxon-signed-rank-test. Sex differences were tested with Mann–Whitney-U-test. Spearman rank order correlations were used to test for consistency of behaviours in two consecutive tests, thereby avoiding the problem of mean level changes due to habituation. Correlations were compared between the three experimental groups or sexes (only for adult animals, sample size of over maturation group was too small to divide by sex) with z-test. Variables with significant correlation coefficients between first and second tests in the ‘short term adult’-group were included in analyses of behavioural types. P-values were adjusted for multiple testing by using a Holm correction [44].

For barrier-test variables ‘activity’ and ‘latency’ repeatability was separately calculated as intraclass correlation coefficient (ANOVA-based repeatability; R_A_) for the three experimental groups. Barrier-test ‘latency’ was log10-transformed to obtain normally distributed data; ‘activity’ was normally distributed. As described in Lessells & Boag [45], R_A_ was based on variance components from a one-way ANOVA with individual as a factor and each variable as dependent variable. Its standard errors were tested with R-package ‘rptR’ following Nakawaga & Schielzeth [46].

To identify possible associations among behavioural variables, we calculated a Spearman rank correlation matrix among all variables from all behavioural tests from the first testing round of the ‘short term adult’-group (see Additional file 1). We computed an agglomerative cluster analysis with the ‘cluster’ package and the ‘agnes’ function of the R statistical environment with ‘manhattan’ clustering with complete linkage, similar to Gyuris et al. [47] and Tremmel & Müller [35]. The results of the cluster analysis are shown by a dendrogram, which lists all of the variables and indicates at what level of similarity any two clusters were joined (‘height’). The height of the link (‘*U*’) represents the distance between the two clusters that contain those objects, i.e. the shorter the *U* the more similar the variables are to each other.

All analyses were carried out with R 2.14 (The R Foundation for Statistical Computing, Vienna, Austria, http://www.R-project.org). Values of p were two tailed throughout and the accepted significance level was p < 0.05.

### Ethical standards

In 2008 all animals were housed and all experiments were conducted under permission of the Landesamt für Natur, Umwelt und Verbraucherschutz Nordrhein-Westfalen (reference number 9.93.2.10.42.07.069). All animals in 2010 and 2011 were captured under permission of the Landesumweltamt Brandenburg (reference number RW-7.1 24.01.01.10). Experiments in 2010 and 2011 were conducted under the permission of the Landesamt für Umwelt, Gesundheit und Verbraucherschutz Brandenburg (reference number V3-2347-44-2011). After the experiments, the animals either stayed in the laboratory for further experiments, or were released at the original trapping site, as specified in the trapping permissions.

## Results

### Consistency over time

All behavioural variables in the ‘short-term adult’ group were highly correlated between the first and second test (Spearman rank correlations, all r_s_ > 0.34, all p < 0.001; details of correlation, mean and SD present in Table 1). The results for the other two groups were less consistent (Table 1). For the animals tested ‘over maturation’ correlations between the first and second round were found in the latency of the barrier test (r_s_ = 0.699, p = 0.001) but not its activity (r_s_ = 0.205, p = 0.43). The ‘long term adult’-voles showed repeatable behaviour in activity of the barrier-test (‘activity’ and ‘crossing frequency’), open-field (‘activity’) and dark–light-test (‘time in light’) (all r_s_ > 0.515, all p <0.05), but not in latency variables of the same tests. In the hole-test, no variable was consistent over time for this group.

**Figure 1:**
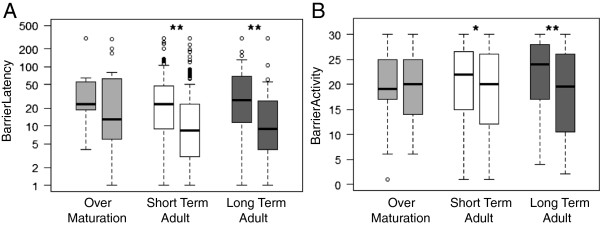
**Comparison between first and second barrier-test within experimental groups of common voles. A)** Barrier Latency [sec], **B)** Barrier Activity [1-0-sampling]. Significant differences in Wilcoxon signed rank test were indicated by stars.

In the barrier-test we found no difference between the three experimental groups (Kruskal-Wallis test by group: latency round1 Chi^2^ = 2.733, df = 2, p = 0.255; round 2 Chi^2^ = 2.9288, df = 2, p = 0.2312; activity round 1 Chi^2^ = 2.9525, df = 2, p = 0.229; round 2 Chi^2^ = 0.8214, df = 2, p = 0.6632) but latency was significantly lower in the second testing round than in the first for the two groups with adult animals if compared within individuals (Wilcoxon signed rank test: short term adult V = 10107, N = 168, p < 0.001; long term adult V = 871, N = 48, p = 0.001; Figure 1A) and activity was reduced (short term adult V = 5638, N = 151, p = 0.021; long term adult V = 595.5, N = 41, p = 0.004), Both was not the case in the animals that were tested over maturation (latency: V = 110, N = 17, p = 0.118; activity: V = 47, N = 17, p = 0.477).

**Figure 2:**
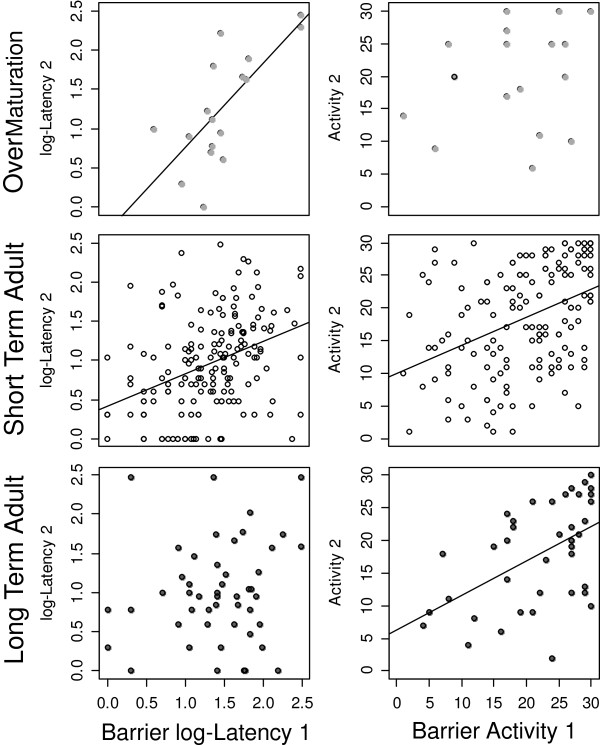
**Correlation between behavioural variables measured in first and second barrier-test.** Latency (left side; range 0–300 sec.) and activity (right side; max. 30 active samples) for each of the three experimental groups of tested common voles are shown. Correlations coefficient (r_s_) and p-values for correlations are represented in Table 1. Lines indicate significant correlations (p < 0.05; adjusted for multiple testing).

Measured latency in barrier-test was repeatable for voles that were tested before and after maturation (R_A_ = 0.63, SE = 0.148, N = 17, p = 0.002) and over one week as adults (R_A_ = 0.27, SE = 0.072, N = 149, p < 0.001), but not for adults that were tested after three months (R_A_ = 0.045, SE = 0.145, N = 41, p = 0.379) (Figure 2). Repeatability of activity in the barrier-test showed the opposite pattern: animals that were tested over adolescence showed no consistent activity (R_A_ = 0.258, SE = 0.23, N = 17, p = 0,145), whereas all tested adults were consistently active, both over one week (R_A_ = 0.387, SE = 0.069, N = 166, p < 0.001) and three months (R_A_ = 0.457, 0.118, N = 48, p < 0.001) (Figure 2). These results were supported by correlations presented in Table 1.

### Consistency over situations

**Figure 3:**
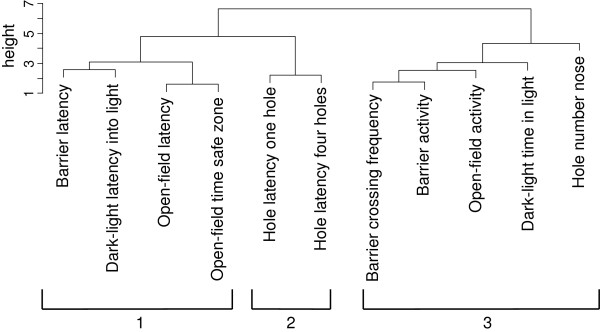
**Dendrogram for the relationships between measured variables in the ‘short term adult’-group (N = 168) according to the cluster analysis.** Height of each *U* represents the distance between two variables and is based on correlation coefficients of Spearman correlation matrix (see Additional file 1). Groupings indicate possible behavioural syndrome structure (agglomerative coefficient 0.64).

On the basis of the correlation matrix (see Additional file 1) and the calculated cluster analysis on the measured variables of the ‘short term adult’-group, three clusters with following variables can be described as possible structures of behavioural syndromes (Figure 3): 1. barrier latency, dark–light latency into light, and open-field latency and time safe zone; 2. hole latency for one and four holes; 3. barrier crossing frequency and activity, open-field activity, dark–light time in light and number of nose-in-hole events. The variables in the first arm represent latency measures. They might be considered as a kind of shy-bold axis of common voles behavioural syndromes, which we have called ‘boldness’. The bolder animals entered the unsafe zone in the open-field and the dark–light-test earlier, jumped earlier over the barrier and stayed in the safe zone of the open-field arena for a shorter time, compared to their shyer conspecifics Close to this cluster is a second arm which includes latencies only of the hole-test. The faster a vole sticks its head through the holes, the more explorative the animal is. Therefore, those latencies are associated with exploration behaviour and are closely related to the boldness variables. Thus, we called both clusters together ‘boldness/exploration’-axes. The third cluster is more uniform, it contains all measured activities of all tests and can therefore be named ‘activity’-cluster. Animals that are active in the open-field are also the more active ones in the barrier-test. They jumped over the barrier more often and spent more time in light.

There were also indications for a connection between boldness and the activity clusters. Common voles which had, for example, short latencies to enter the unsafe zone in the open-field, did also jump at the barrier at higher frequency, i.e. show higher activity (Spearman rank correlation r_s_ = -0.327, N = 164, p < 0.01).

### Sex differences

Sex differences were found only in barrier and open-field activity where male common voles where more active than females (barrier activity: males 21 ± 8, females 19 ± 8 active samples; Mann–Whitney-U = 2249.0, N = 151, p = 0.026; open-field activity: males 22 ± 7, females 19 ± 8 active samples, Mann–Whitney-U = 2602.5, N = 164, p = 0.014). In all other behaviours males and females behaved equally. Comparison of Spearman correlations that were calculated separately for adult males and females showed no difference in consistency over time between sexes (z-test, all p > 0.3).

## Discussion

Our results demonstrate the presence of behavioural syndromes in common voles (*Microtus arvalis*). We showed that behaviour in different tests was consistent over time (Figure 2) and over situations (Figure 3).

Since rank order of scores is maintained across contexts in adult animals, i.e. all activities and according latencies form one arm of the dendrogram (Figure 3), contextual generality was high [24]. With a combination of activity and boldness/exploration as dimensions of behavioural syndromes we obtained results very similar to studies in voles and other small mammals. Lantová *et al*. [38] also found a connection between activity and boldness in different behavioural tests in a captive common vole population. Similar axes can also be seen in grey mouse lemurs (*Mircrocebus murinus*) [32] and Siberian chipmunks (*Tamias sibiricus*) [48] in wild populations. Other vole species showed consistent individual differences in activity (meadow vole (*Microtus pennsylvanicus*[49]; root voles *M. oeconomus*) [50]) like adult animals in our study. Therefore, it can be assumed that boldness and activity are very common personality axis in small mammals.

As predicted, the length of the interobservation interval had an influence on the repeatability of behaviours in common voles. Animals that were tested over intervals of three months, over maturation or as adults, showed less consistent behaviours than animals tested twice during one week (Table 1). Surprisingly, activity and boldness/exploration behaviour differed in their behavioural consistency, depending on the age of the tested animal. This may possibly be explained by differences in experiences, their expected life span, or developmental change such as maturation.

Young voles tested over maturation lived in stable captive conditions before and over the experiment. They had stable latencies while exploring the barrier-test but do not show repeatable activity. It is possible that the laboratory environment with limited space for experiencing or exercising movement did not stimulate the formation of stable activity types. We compared captive bred animals with wild caught ones in this study by the reason that adequate age estimation is not possible in wild-caught voles and immature young voles were difficult to trap in nature. Due to habituation to captive conditions and lower diversity of situations one could expect that captive animals show even more stable behaviours [51] but Bell et al. [28] found repeatability of behaviour was higher when measured under natural conditions. Besides, another reason for the missing consistency over time is that some effects cannot be detected with small sample sizes. To proof this result on inconsistency in boldness in more natural conditions (e.g. in monitored outdoor enclosures) and with a larger sample size would be challenging for future studies.

Adult voles captured from the wild and maintained in semi-wild outdoor enclosures, showed repeatability in boldness/exploration behaviour only over a short period of time, but less so over three months. In contrast activity patterns were stable, which is surprising because activity has been reported as one of the least repeatable behaviours [28] (but see [34]). It could be that exploration and boldness can be adapted more easily to current environmental conditions like predator presence or food availability, which may have differed between conditions in the wild, which animals experienced prior to the first testing and in the enclosures, prior to the second testing. Experiences can either enhance stability by reinforcing a package of traits, or experiences may modify behavioural types [3]. In a small prey animal, like the common vole, this flexibility could be very important to survive in the wild where force trade-offs between, for instance, exploring resources and avoiding predators. This flexibility is less important in the laboratory where conditions were stable. We suggest that activity patterns were developed after maturation as we could not see them in young animals but in adults. Studies on killifish (*Kryptolebias marmoratus*) [52] and Sibirian dwarf hamsters (*Phodopus sungorus*) [34] showed also an age effect on behavioural types and developmental flexibility while damselflies (*Lestes congener*) show developmental consistency in boldness and activity pertaining through metamorphosis [53].

Current theoretical models discuss that differences in state in combination with state-dependent behaviour can explain stable differences in behavioural traits [54]. For example, animals should differ in their risk-taking behaviour if they do either focus on current or on future reproduction. Individuals with expected low future reproductive success (i.e. low asset) should take more risk as they have not much to lose [32,54,55]. Meanwhile, in our study, juvenile voles, that should have high assets to protect, did not have lower boldness or higher activity scores compared to adult animals with expected lower future assets (Figure 1). Studies on field crickets (*Gryllus integer*) [21] and grey mouse lemurs [32] support that a trade-off between current and future reproduction can lead to personality variation. To investigate why behaviour of common voles is not adjusted like predicted in the asset protection theory, the differences in behaviour between young and older voles should be observed in natural populations with fluctuating densities and active competition.

The differences in differential consistency in the three experimental groups suggest that the link of activity and boldness/exploration can be decoupled during ontogeny. This makes sense when environmental conditions experienced by juveniles differ substantially from those experienced by adults [3], particularly in traits that are more sensitive to the environment (e.g. behavioural traits like activity or boldness) compared to morphological or physiological traits [28]. In voles, annual population dynamics with different conditions depending on the season of birth are typical: Animals that were born in spring/summer mature at an age of weeks, whereas individuals born in late summer/autumn overwinter as immatures and start reproduction in the following spring at an age of months [56-59]. During seasons not only environmental conditions change (e.g. density, food availability) but also asset protection should play a role in development of behavioural traits, like discussed above and elsewhere [60]. Therefore, the importance of studies in natural conditions should not be neglected. It is likely that especially young individuals could show different development of behavioural traits in unstable or heterogeneous environments. Environmental conditions affect both life history [61] and behavioural type expression [15,62], and future studies should consider both.

Male common voles were more active in two of four tests than females. The voles’ breeding system with promiscuously mating females may explain this difference as discussed in Eccard & Herde [60]. Differences in consistency over time between the sexes were not found although this would not be unexpected as this was found in two bird species [12,63].

## Conclusions

Common voles showed consistent individual differences in their behaviour. Over short periods, repeatability as well as contextual generality was high. Over longer periods, long in relation to the animals’ life span, stability of traits may be dependent of life stage of the animal but also external factors like stability of the surrounding environment. The research on animal personality and behavioural syndromes requires longitudinal studies (e.g. capture-mark-recapture) to follow the variation in behaviour and its consistency over a life span under natural conditions [13].

## Abbreviations

PIT: Passive integrated transponder; ANOVA: Analysis of Variance; RA: ANOVA-based repeatability; SE: Standard error; rs: Spearman rank correlations coefficient; SD: Standard deviation.

## Competing interests

The authors declare that they have no competing interests.

## Authors’ contributions

AH and JAE designed the study. AH performed the research, analysed the data and wrote the manuscript. Both authors read and approved the final paper.

## Supplementary Material

Additional file 1 Spearman correlation matrix of first testing round for all measured variables in the ‘short term adult’-group. Click here for file
