# Supplementary material for: Consistency in boldness, activity and exploration at different stages of life
Source: BMC Ecol. 2013 Dec 7;13:49. doi: 10.1186/1472-6785-13-49 (PMC3878951; doi:10.1186/1472-6785-13-49)
Supplement: Additional file 1 — Spearman correlation matrix of first testing round for all measured variables in the ‘short term adult’-group. [file 1472-6785-13-49-S1.pdf]

Additional file

Additional file: Spearman correlation matrix of first testing round for all measured variables in the 'short term adult'-group

			**Barrier**			**Open-field**			**Dark–light**		**Hole**	
			**Latency**	**Crossing frequency**	**Activity**	**Latency unsafe zone**	**Time in safe zone**	**Activity**	**Latency into light**	**Time in light**	**Latency one hole**	**Latency four holes**
**Barrier**	Crossing frequency	**r_s**	**−0.268**									
		**p**	**0.08**									
		**N**	**168**									
	Activity	**r_s**	−0.225	**0.521**								
		**p**	0.84	**<0.01**								
		**N**	151	**151**								
**Open-Field**	Latency unsafe zone	**r_s**	0.230	**−0.327**	−0.228							
		**p**	0.48	**<0.01**	0.84							
		**N**	164	**164**	147							
	Safe zone	**r_s**	−0.037	−0.196	−0.215	**0.572**						
		**p**	1.0	1.0	1.0	**<0.01**						
		**N**	164	164	147	**164**						
	Activity	**r_s**	−0.200	0.222	**0.351**	−0.248	**−0.309**					
		**p**	1.0	0.67	**<0.01**	0.23	**0.01**					
		**N**	164	164	**147**	164	**164**					
**Dark–light**	Latency into light	**r_s**	0.174	**−0.328**	−0.169	**0.324**	0.169	−0.209				
		**p**	1.0	**<0.01**	1.0	**<0.01**	1.0	1.0				
		**N**	166	**166**	149	**164**	164	164				
	Time in light	**r_s**	−0.136	0.200	0.188	−0.200	−0.092	0.192	**−0.530**			
		**p**	1.0	1.0	1.0	1.0	1.0	1.0	**<0.01**			
		**N**	166	166	149	164	164	164	**166**			
**Hole**	Latency one hole	**r_s**	0.128	−0.236	−0.106	0.057	0.040	0.040	0.188	−0.261		
		**p**	1.0	0.97	1.0	1.0	1.0	1.0	1.0	0.40		
		**N**	132	132	132	131	131	131	132	132		
	Latency four holes	**r_s**	−0.014	−0.080	−0.198	−0.036	−0.012	−0.002	0.003	−0.063	**0.399**	
		**p**	1.0	1.0	1.0	1.0	1.0	1.0	1.0	1.0	**<0.01**	
		**N**	132	132	132	131	131	131	132	132	**132**	
	Number nose	**r_s**	−0.089	0.145	0.075	0.055	0.045	0.077	−0.035	0.045	**−0.550**	**−0.490**
		**p**	1.0	1.0	1.0	1.0	1.0	1.0	1.0	1.0	**<0.01**	**<0.01**
		**N**	132	132	132	131	131	131	132	132	**132**	**132**

P-values adjusted for multiple testing with Holm correction. Significant correlations are in bold ($p < 0.05$).
